# It's all in the past: temporal-context effects modulate subjective evaluations of emotional visual stimuli, regardless of presentation sequence

**DOI:** 10.3389/fpsyg.2015.00367

**Published:** 2015-04-07

**Authors:** Kristína Czekóová, Daniel J. Shaw, Eva Janoušová, Tomáš Urbánek

**Affiliations:** ^1^Behavioral and Social Neuroscience Research Group, Central European Institute of Technology, Masaryk UniversityBrno, Czech Republic; ^2^Faculty of Medicine, Institute of Biostatistics and Analyses, Masaryk UniversityBrno, Czech Republic; ^3^Institute of Psychology, Academy of Sciences of the Czech RepublicBrno, Czech Republic

**Keywords:** emotion, temporal context, presentation sequence, assimilation effect, contrast effect

## Abstract

The aim of this study was to investigate if and how temporal context influences subjective affective responses to emotional images. To do so, we examined whether the subjective evaluation of a target image is influenced by the valence of its preceding image, and/or its overall position in a sequence of images. Furthermore, we assessed if these potentially confounding contextual effects can be moderated by a common procedural control: randomized stimulus presentation. Four groups of participants evaluated the same set of 120 pictures from the International Affective System (IAPS) presented in four different sequences. Our data reveal strong effects of both aspects of temporal context in all presentation sequences, modified only slightly in their nature and magnitude. Furthermore, this was true for both valence and arousal ratings. Subjective ratings of negative target images were influenced by temporal context most strongly across all sequences. We also observed important gender differences: females expressed greater sensitivity to temporal-context effects and design manipulations relative to males, especially for negative images. Our results have important implications for future emotion research that employs normative picture stimuli, and contributes to our understanding of context effects in general.

## Introduction

Our evaluation of events is influenced profoundly by the context in which we encounter them (Barrett and Kensinger, 2010; Anderson et al., 2011; Barrett et al., 2011). A long history of psychological research has demonstrated that our perception and attitude towards a given stimulus is altered by preceding or simultaneously presented stimuli. Evidence for such context effects has accumulated in a variety of literatures, such as those concerning perception (Brown, 1953; Parducci, 1995; Ricci and Chatterjee, 2001; Sarris, 2006), attitude measurement (Wittenbrink et al., 2001; Blair, 2002), social cognition (Sherif and Hovland, 1961; Herr, 1986; Bless and Schwarz, 2010; Todd et al., 2011), and evaluative judgments (e.g., Zellner et al., 2003; Cogan et al., 2013). In particular, two kinds of context effects are reported consistently: contrast and assimilation. The former refers to diverging evaluations of two similar stimuli presented successively, while the latter involves the comparable evaluation of a target and its preceding stimulus despite their dissimilarity (Zellner et al., 2003).

Such *temporal-context effects* could present an important confound in research that seeks to quantify objectively an individual's affective response to emotional stimuli, especially since subjective evaluations (i.e., ratings) are the standard measurement for stimulus differentiation and selection. Surprisingly, however, the influence of temporal context on subjective emotional experience has not yet been addressed comprehensively. The present study set out to investigate the degree to which temporal context influences our experience of emotional stimuli, and to identify any variables that might modify such confounding effects.

Previous research suggests that temporal-context effects are indeed present during emotion processing (e.g., Flaisch et al., 2008a,b; Larsen and Norris, 2009; Barrett and Kensinger, 2010). A series of studies have demonstrated that electro-cortical, autonomic and behavioral responses elicited by positive, negative, and indifferent^1^ images differ when they are preceded by emotional (positive and negative) compared with indifferent verbal descriptions (i.e., appraisal frames; Foti and Hajcak, 2008; MacNamara et al., 2009, 2011; Wu et al., 2012). Such relativity of subjective evaluations of target images as a function of their preceding stimuli can be also inferred from studies that investigate affective priming: target emotional images are processed more efficiently (i.e., faster) when preceded by a prime with congruent relative to incongruent valence (e.g., Herring et al., 2011). The opposite is true for primes with incongruent valence, whereby interference effects can be observed at the neurophysiological (Klauer and Musch, 2003) and behavioral level (Herring et al., 2011).

Importantly, however, the majority of these studies only contrasted emotional with indifferent stimuli, and focused predominantly on psycho- and neuro-physiological responses. Either no information on subjective evaluations of target images was acquired due to the method of presentation (e.g., short stimulus durations; Flaisch et al., 2008a), or it is limited to basic contrasts between specific valence categories (i.e., positive, negative, and indifferent; Schupp et al., 2013) that are unlikely to detect subtle differences in evaluations. To the best of our knowledge, only Fujimura et al. (2013) investigated the effect of temporal context in psychophysiological and subjective responses to emotional stimuli, and performed contrasts between *all* valence groups. These authors report context-related modulations in both levels of measurement, especially when the temporal context involved negative preceding stimuli.

Research findings are far from consistent, however; other research indicates that emotional images are processed independently of the preceding context (Pastor et al., 2008; Schupp et al., 2013). Specifically, some studies report that while indifferent stimuli are vulnerable to context manipulations (Pastor et al., 2008; MacNamara et al., 2009), the processing of emotional stimuli is driven purely by the valence of the target image rather than its temporal context (Pastor et al., 2008; Schupp et al., 2013).

Moreover, it is generally assumed that any potentially confounding effects of temporal context are controlled by using a random presentation sequence. Indeed, this is the approach taken by many studies, usually with a constraint of no more than two or three stimuli of the same valence category presented successively to avoid mood induction (e.g., Bradley et al., 2001a; Bublatzky et al., 2013). The assumption that random presentation sequences minimize the confounding influence of temporal-context effects has not yet been tested empirically, however, nor is it clear what form of randomization is optimal—a sequence randomized for each participant individually, or the same randomized sequence presented to all participants. Determining the effectiveness of different presentation sequences vis-a-vis their efficacy in accounting for temporal-context effects should therefore provide valuable information for the design of future experimental procedures.

An additional contextual factor likely to influence subjective evaluations is the number of stimuli encountered prior to a target. Studies that present the same stimuli repeatedly report habituation, whereby subjective ratings of valence and arousal become less extreme over time (Codispoti et al., 2006; Wendt et al., 2012). On the other hand, repeated presentation of the same valence category—and for negative images especially—is reported to result in an increase in affective responses (“sensitization”; Bradley et al., 1996). Since studies vary considerably with respect to the number of stimuli presented, the duration of their presentation, and the overall length of the experimental procedure, defining more precisely the time-course of subjective evaluations—and any temporal-context effects—would also offer valuable recommendations for future emotion research. Furthermore, since both normative valence and arousal ratings are considered when selecting stimuli for experimental studies, future emotion research will benefit from understanding how contextual influences modulate each dimension separately.

Finally, large inter-individual variability in the evaluation of emotional stimuli (see Hamann and Canli, 2004), and subjective emotional experiences in general (Kuppens et al., 2013), should be considered when investigating context effects. Gender differences in the perception of emotionally evocative stimuli are reported consistently (Bradley et al., 2001b; Gard and Kring, 2007; Lithari et al., 2010), with evidence suggesting a greater sensitivity toward highly arousing negative stimuli in females (Gohier et al., 2013). Further, emotion regulation is proposed to be a more automatic and unconscious process in males compared with females (Barrett et al., 2000). A comprehensive assessment of temporal-context effects must therefore explore if and how this potential confound influences these two groups differently.

We explored the potential influence of these factors on the subjective evaluation of emotional and indifferent images. First we examined the influence of *Temporal Context* directly, as defined by the valence of the image preceding a target stimulus. Subjective evaluations were measured by both valence and arousal ratings. We predicted that preceding pictures would influence the evaluation of subsequent stimuli to a larger degree when they were emotional, and such modulation should emerge in both dimensions. Considering their motivational and/or biological significance, we expected negative target images to be influenced much less by temporal context. For the same reason, we hypothesized that indifferent images would be affected to a much larger extent. In light of affective priming research, we also expected that context-target combinations of the same valence category (namely, positive-positive and negative-negative) will lead to congruency effects; that is, the behavioral response to an emotional target of the same valence as its preceding image will be potentiated. Secondly, to assess the time-course of any temporal-context effects, and the habituation or sensitization effects as they unfold throughout the course of an experiment, we examined whether subjective evaluations differed according to the position of target stimuli within a sequence (referred to herein as *Trial*). Lastly, we investigated whether these potential confounds are modulated by *Gender* and/or presentation sequence. Given the reported differences in emotion experience and response between males and females, we hypothesized that females would be influenced by temporal context to a larger degree than males, especially with regard to negative context. Four different presentation sequences were compared in terms of their ability to minimize these confounding effects. On the basis of current assumptions, we expected the least amount of temporal-context effects to emerge from a random presentation.

## Methods

### Participants

The sample consisted of 294 participants (96 males) who were allocated randomly to each of four groups defined by the sequence in which images were presented. The mean age of the sample was 24.46 [standard deviation (SD) = 6.97] years. There was no significant difference between males and females with respect to age in any of the presentation sequences: *U* = 8339.00, *z* = −1.18, *p* = 0.24, and all groups contained a similar male–female ratio: χ^2^(3) = 2.57, *p* = 0.46. The sample comprised students and associates of Masaryk University, Czech Republic. All had normal or corrected-to-normal vision, and reported no psychological or psychiatric disorders. Explicit informed consent was acquired from each participant before the procedure began. The study was approved by the Ethics Board of the Institute of Psychology, Academy of Sciences of the Czech Republic.

### Stimuli

The stimuli comprised 120 color photographs^2^ from the International Affective Picture System (IAPS; Lang et al., 2005). The stimuli were defined as positive, negative and indifferent (mean valence = 7.42, 2.38, and 5.25, respectively), using a random selection from the available IAPS set within the limits for each valence category. This resulted in a total of 40 stimuli per valence category.

To investigate if and how *Temporal-Context* effects manifest in each presentation sequence, participants were allocated to one of four groups defined by the sequence in which stimuli were presented: *Random_FIX_*—The same randomized sequence^3^ presented to all participants, in which no more than three pictures of the same valence category [negative (N), positive (P), and indifferent (I)] were presented successively; *Random*—a randomized sequence generated for each individual, again containing no more than three pictures of the same valence category presented successively; *Fixed_INC_*—a fixed sequence in which each image was presented in order of increasing valence, within a set sub-sequence repeated 10 times (N-I-P-P-N-I-I-P-N-N-I-P; the most negative and least positive images presented at the beginning); *Fixed_DEC_*—a fixed sequence in which images were presented in the reverse set order of gradually decreasing valence (P-I-N-N-P-I-I-N-P-P-I-N). In all sequences, each of the 120 images was presented only once. With the exception of *Random*, the same sequence was presented to all participants.

All possible combinations of preceding and target image valence groups (referred to as context-target combinations) were calculated for sequences *Random_FIX_* and *Random*: negative, positive, and indifferent target images were preceded by all three context valences (i.e., N-N, P-N, I-N; N-P, P-P, I-P; and N-I, P-I, I-I). As a result of the design, however, some context-target combinations were not presented in sets *Fixed_INC_* and *Fixed_DEC_*; namely N-P, I-N, and P-I for the former, and P-N, I-P, and N-I for the latter. Therefore, contrasting context-target combinations were missing for negative and positive targets in these respective sequences.

To assess whether or not overall sequence position influenced subjective evaluation of the target image, we examined the effect of their position relative to the other targets within that valence category (*Trial*; i.e., 1–40).

The experimental procedure was written entirely in HTML, and participants completed the task at their own pace via an intranet website. Pictures were presented at a resolution of 1024 × 768. They were required to rate each picture on two nine-point scales before the subsequent trial began—one for valence and the other arousal. The Self-Assessment Manikin (SAM; Bradley and Lang, 1994) was employed to rate the stimuli. The dimension of dominance was not included in the study due to the relatively small amount of variance it explains (Bradley and Lang, 1994). The participants were naïve as to the length and/or number of images presented.

### Statistical analyses

Statistical analyses were performed using SPSS 22 Software. Generalized Linear Mixed Models (GLMMs) were used to investigate the effects of *Temporal Context* on subjective evaluations of target images (i.e., valence and arousal ratings). This analytical technique also allowed us to investigate whether there was an effect of *Trial* in each sequence; specifically, whether or not ratings change throughout the course of each sequence, and how this interacts with any *Temporal-Context* effects. Ratings of positive, negative, and indifferent images were analyzed separately for each sequence; first for males and females combined, and then for each gender separately.

Preliminary analyses confirmed considerable variability between individuals with respect to subjective evaluation. To account for this, our GLMMs included a random intercept and *Trial* effect nested within participant. The models were developed in a step-down approach (West et al., 2006), with non-significant variables omitted at each step (Andersen and Skovgaard, 2010). The final models included *Temporal Context* and *Trial* as fixed effects, and, when significant, their interaction term. For all analyses, *α* = 0.05 and multiple-comparison correction was performed using the Bonferroni method. Only participants who rated the entire stimulus set were included in the analyses.

## Results

**Figure 1:**
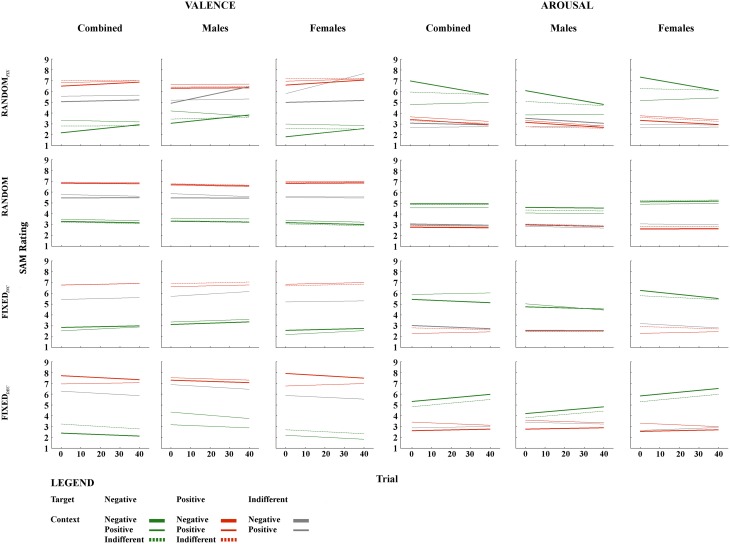
**Fixed effects of temporal context for valence and arousal**. The figures are plotted using the intercept and slope estimates given in Tables 1–4; specifically, the intercepts given in the *Intercept* and *Temporal Context* columns, and the slope estimates given in the *Trial* and (where the interaction term was significant) Temporal Context*Trial columns. *Abbreviations: Random_FIX_*, The same randomized sequence presented to all participants; *Random*, A sequence randomized for each participant independently; *Fixed_INC_*, A fixed sequence presented to all participants, in which valence increased gradually throughout but no more than three stimuli of the same valence category were presented successively; *Fixed_DEC_*, The reverse of *Fixed_INC_*, such that valence decreased gradually throughout; Trial, succession of individual temporal context-target combinations over the course of the task. *Note:* Trial numbers represent the relative positioning of target stimuli within a sequence, rather than their actual positioning over the course of 120 images.

To examine the efficacy of each sequence to control for any effects of *Temporal Context* and/or *Trial*, we report the results for each sequence separately. We describe the ratings for each dimension first for the combined sample, and then for each gender separately. For the sake of brevity, we discuss only those findings related most closely to our research question; we refer the reader to Tables 1, 2, and Figure 1 for other, more specific patterns of results.

**Table 1:**
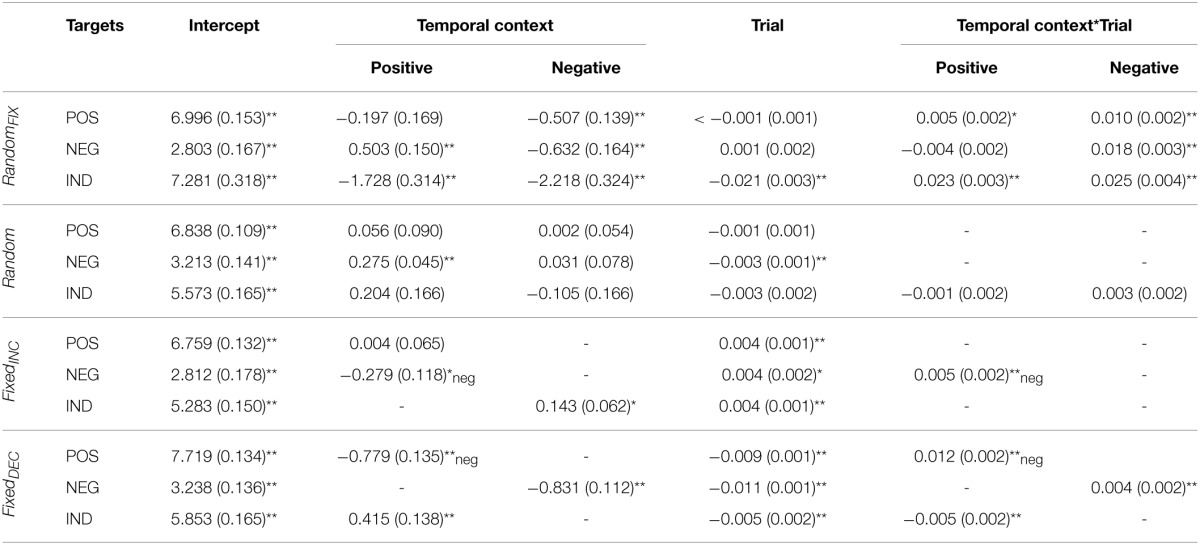
**Fixed effects parameter estimates for valence ratings, for the entire sample combined**.

Tables 1-4. Parameter estimates for each category of target image according to its temporal context. For sequences with all possible context-target combinations (i.e., Random_FIX_ and Random), an indifferent temporal context was used as a reference—Intercept and Trial columns present, respectively, the absolute intercept and slope for each target category when preceded by an indifferent image; and the Temporal Context and Temporal context^*^Trial columns present, respectively, the relative change in the intercept and slope estimates when the target category is preceded by a positive or negative image. For sequences in which certain context-target combinations were not possible (see text for details), targets preceded by negative images serve as the reference. The latter comparisons are indicated by _neg_. Note: Values present means (± standard errors); **p < 0.01; *p < 0.05.

**Table 2:**
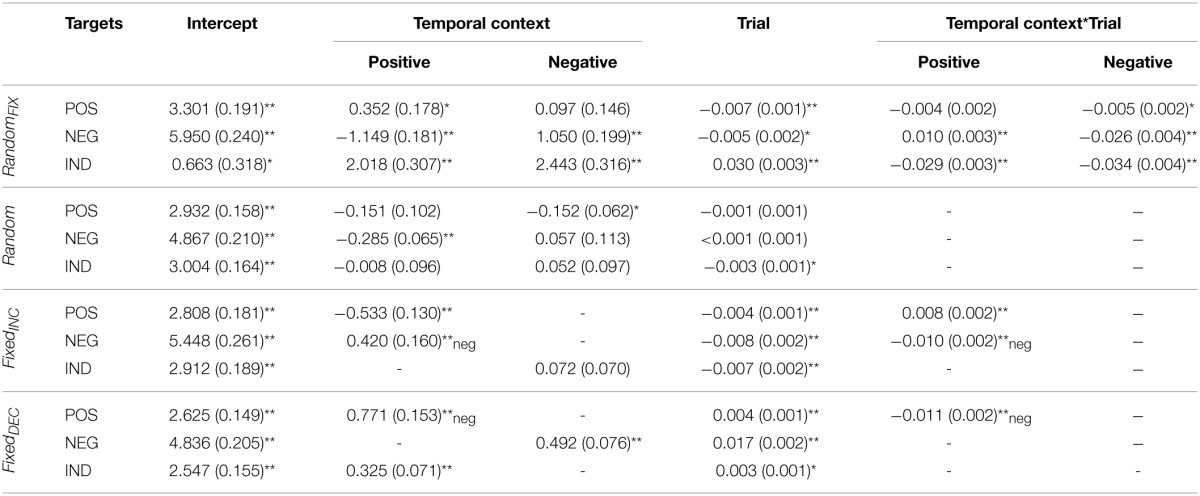
**Fixed effects parameter estimates for arousal ratings, for the entire sample combined**.

### Random_FIX_

When collapsing across gender, *Temporal-Context* effects were present in this sequence for subjective evaluations of all three target categories; valence ratings of negative [*F*_(2, 2884)_ = 29.39, *p* < 0.001], positive [*F*_(2, 2884)_ = 6.68, *p* < 0.01], and indifferent [*F*_(2, 2808)_ = 23.57, *p* < 0.001] target images were influenced by the valence category of their immediately preceding image. These effects tended to be transient, however, as indexed by *Temporal Context*-by-*Trial* interactions (see Table 1). Arousal ratings for negative and indifferent targets were also influenced by *Temporal Context* [*F*_(2, 2884)_ = 76.64, *p* < 0.001; and *F*_(2, 2808)_ = 29.80, *p* < 0.001; respectively].

A *Trial* effect revealed that arousal ratings of emotional images decreased gradually during this presentation sequence; this was true particularly for negative [*F*_(1,120.55)_ = 33.39, *p* < 0.001] and positive images [*F*_(1,97.20)_ = 87.03, *p* < 0.001], which demonstrated habituation.

The *Temporal Context*-by-*Trial* interaction revealed that negative target images were rated as significantly more pleasant and less arousing when seen after a positive relative to indifferent context, respectively, and less pleasant and more arousing after seeing negative compared with indifferent images, respectively, at the beginning of this presentation sequence. In other words, these confounding effects diminished with increasing trials. Positive target images were evaluated as less pleasant when following a negative relative to an indifferent image, and more arousing when seen after a positive compared with an indifferent, respectively, but again these effects did not hold throughout the entire task. Finally, indifferent target images were rated as less pleasant and more arousing when presented after emotional images regardless of their specific valence category, but these influences were also temporary.

As can be seen from Tables 3, 4, no major gender differences existed with respect to *Temporal Context* or *Trial* effects on valence and arousal ratings for indifferent target images. Females were more sensitive to negative contexts when rating valence, however, and their arousal ratings for negative targets did not decrease gradually to the same extent observed in males. Females' valence ratings of positive images were also influenced transiently by the preceding image. Regarding arousal ratings of positive targets, females demonstrated separate *Temporal Context* and *Trial* effects throughout the task, while males were influenced by *Temporal Context* only at the beginning of the sequence. In general, females seemed to be more affected by negative preceding images compared with males, especially at the beginning of this presentation sequence (see Figure 1).

**Table 3:**
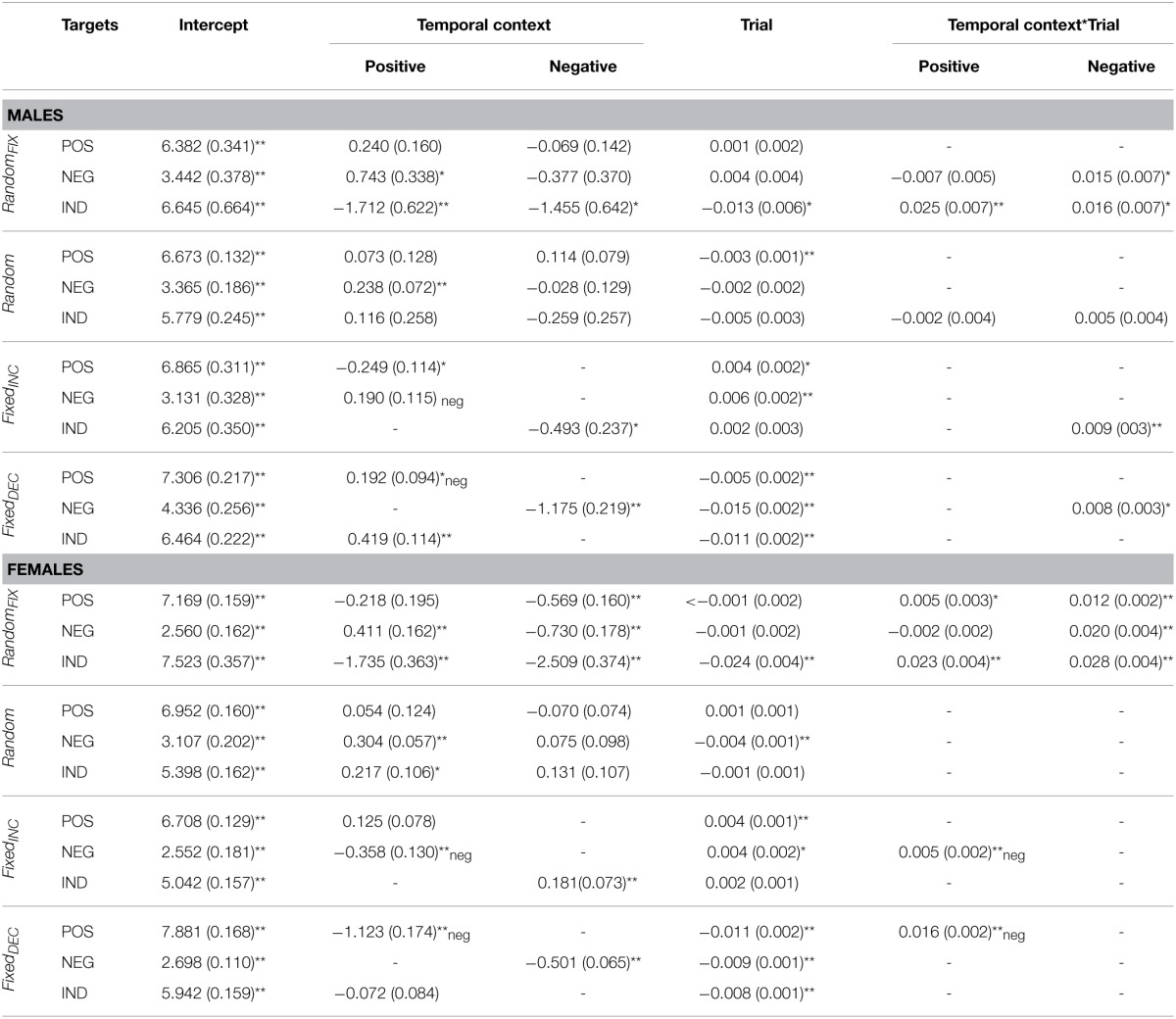
**Fixed effects parameter estimates for valence ratings according to gender**.

**Table 4:**
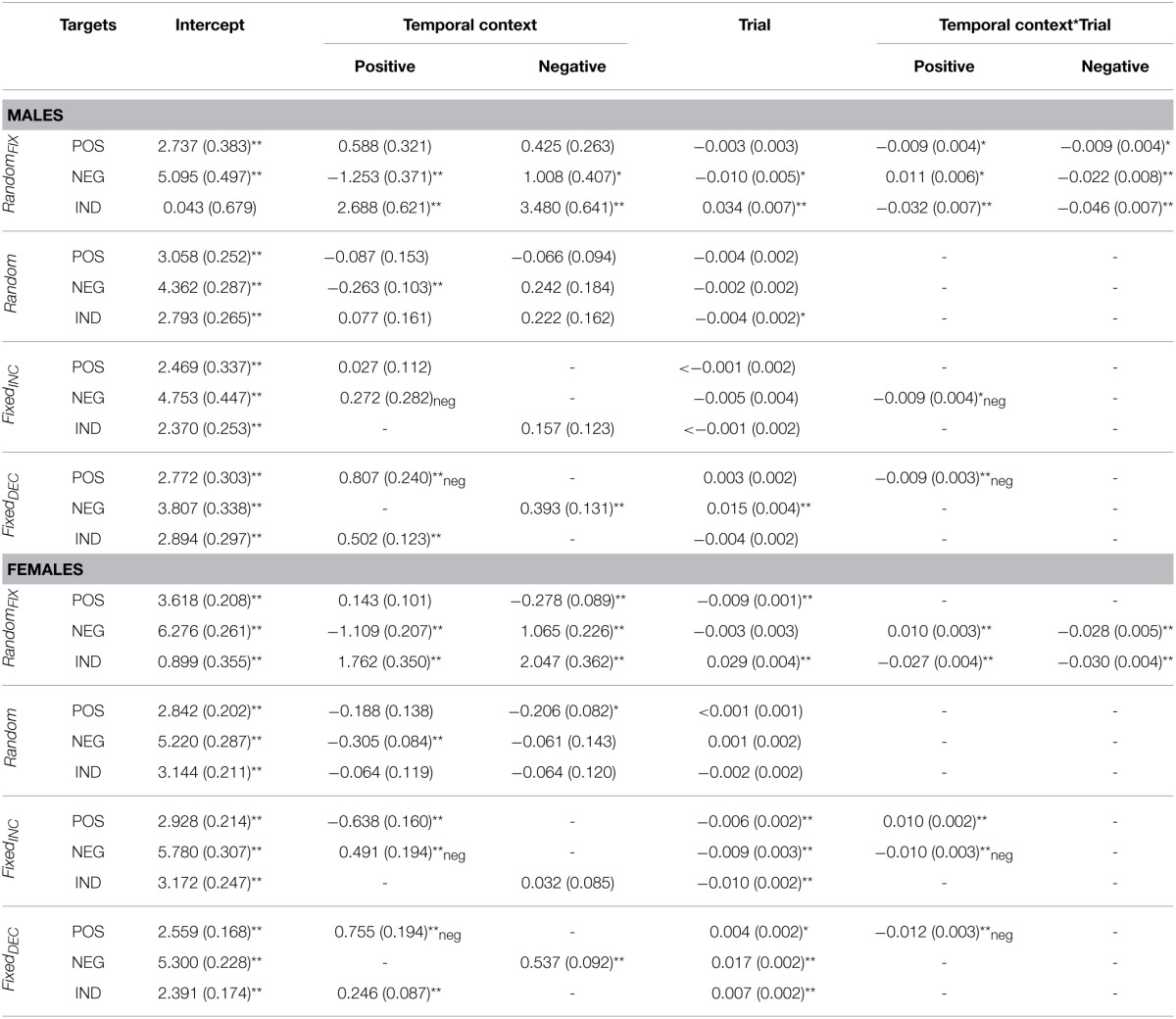
**Fixed effect parameter estimates for arousal ratings according to gender**.

### Random

As expected, *Temporal-Context* effects were relatively sparse for this presentation sequence. When they were present, however, they manifested consistently throughout the entire task. *Temporal Context* and *Trial* effects influenced valence ratings of negative target images significantly [*F*_(2, 2987.76)_ = 19.62, *p* < 0.001; *F*_(1, 77.48)_ = 12.83, *p* < 0.01, respectively]; negative targets were rated as more pleasant when preceded by a positive in contrast to both negative and indifferent preceding images, and their ratings decreased gradually throughout the sequence. Arousal ratings of negative targets were also altered significantly by their preceding stimulus [*F*_(2, 2988.05)_ = 11.31, *p* < 0.001]; they were rated as less arousing when preceded by a positive relative to negative and indifferent images. No effects were detected for valence ratings of positive target images, but their arousal ratings were influenced by *Temporal Context* [*F*_(2, 2957.49)_ = 3.23, *p* < 0.05]; positive targets were rated as less arousing when seen after negative compared with indifferent images. Arousal ratings also became gradually lower for indifferent target images [*F*_(1, 75.58)_ = 5.00, *p* < 0.05].

Gender differences were observed in the valence ratings of negative targets; namely, females were more sensitive to the *Trial* effect than males. Females were also influenced by *Temporal Context* while rating the valence of indifferent targets. The only difference with respect to arousal was detected for positive targets, which were influenced by negative preceding images in females but not males (see Tables 3, 4).

### Fixed_INC_

In this sequence, *Temporal Context* exerted an effect on valence ratings for negative and indifferent target images [*F*_(1, 2514)_ = 5.62, *p* < 0.05; *F*_(1, 2583)_ = 5.28, *p* < 0.05, respectively], and on arousal ratings for negative and positive target images [*F*_(1, 2514)_ = 6.89, *p* < 0.001; *F*_(1, 2582)_ = 16.82, *p* < 0.001, respectively]. Indifferent target images were evaluated as more pleasant when following negative relative to indifferent preceding images. Furthermore, a *Temporal Context*-by-*Trial* interaction was found for negative targets on both dimensions [valence: *F*_(1, 2514)_ = 9.58, *p* < 0.01; arousal: *F*_(1, 2514)_ = 18.81, *p* < 0.001], indicating that these images were rated as less pleasant and more arousing when following positive rather than negative images at the beginning of the sequence, respectively. As expected, we observed an effect of *Trial* on valence ratings that followed the intended pattern; they increased gradually for negative [*F*_(1, 80.02)_ = 22.21, *p* < 0.001], positive [*F*_(1, 67.17)_ = 14.00, *p* < 0.001] and indifferent targets [*F*_(1, 67.00)_ = 13.90, *p* < 0.001]. Correspondingly, arousal ratings for negative and indifferent target images decreased with the completion of more trials [*F*_(1, 80.26)_ = 51.72, *p* < 0.001; and *F*_(1, 67.00)_ = 20.14, *p* < 0.001; respectively].

With respect to gender, negative and positive preceding images (*Temporal Context*) had a differential influence on valence ratings. First, relative to males, females evaluated indifferent targets as more pleasant after seeing negative relative to indifferent preceding images. Secondly, positive relative to indifferent preceding images resulted in temporarily less pleasant ratings of negative targets in females relative to negative preceding images. Furthermore, females were affected by *Temporal Context* while rating the arousal of positive and negative target images at the beginning of the sequence, while the same was not true for males. Lastly, *Trial* effects remained significant for arousal ratings of all target categories for females, but not for males—there was only *Temporal context*-by-*Trial* interaction in case of Negative target images.

### Fixed_DEC_

As shown in Table 1 and illustrated in Figure 1, significant *Temporal Context, Trial*, and *Temporal Context*-by-*Trial* interactive effects were confirmed for valence ratings of all three target categories: [interactions: negative: *F*_(1, 2810)_ = 7.22, *p* < 0.01; positive: *F*_(1, 2736)_ = 41.32, *p* < 0.001; indifferent: *F*_(1, 2810)_ = 7.71, *p* < 0.01]. For arousal, a significant interaction applied only to ratings of positive target images [*F*_(1, 2736)_ = 26.68, *p* < 0.001]. Concerning the *Trial* effect, negative and indifferent targets were rated gradually more unpleasant [*F*_(1, 100.51)_ = 73.69, *p* < 0.001; *F*_(1, 116.70)_ = 49.86, *p* < 0.001] and arousing [*F*_(1, 73.10)_ = 102.68, *p* < 0.001; *F*_(1, 73.00)_ = 5.63, *p* < 0.05]. Furthermore, valence ratings of positive targets decreased [*F*_(1, 96.28)_ = 6.92, *p* < 0.05] and arousal ratings increased with more trials completed. These latter *Trial* effects were significant only at the beginning of the sequence, however.

More specifically, negative target images were rated as less pleasant and more arousing when they followed a negative compared to indifferent image. This effect of *Temporal Context* on arousal ratings remained significant throughout the entire task. Positive target images were evaluated as less pleasant and more arousing when they followed positive rather than negative pictures, but these effects diminished with more trials completed. Indifferent targets were rated as more pleasant and more arousing when presented after positive relative to indifferent images. The influence on valence ratings was significant only at the beginning of the sequence.

No major gender differences were observed for valence ratings of negative and indifferent target images and arousal ratings of negative and positive target images. In contrast, a gender effect was revealed in the *Temporal-Context* effect for positive target images: females rated positive targets as more unpleasant toward the beginning of this sequence. With respect to arousal, a difference was found in ratings of indifferent targets, which were gradually increasing in females relative to males.

## Discussion

The aim of our study was to investigate if and how subjective experiences of emotional visual stimuli vary according to the context in which they appear. To this end, we examined whether subjective evaluations of target images are influenced by the valence of their immediately preceding image and/or their position within a presentation sequence (temporal-context and trial effects, respectively). To assess this influence in a comprehensive manner, we examined whether these factors exert an influence in four different presentation sequences, and contrasted all possible context-target combinations; specifically, we applied general linear mixed models (GLMMs) to each sequence independently. Additionally, by treating males and females separately we were able to investigate the potential modulation of these contextual effects by gender. In the sections that follow, we present our findings according to these three main themes.

Firstly, and perhaps most importantly, our data provide evidence that temporal-context effects are present in some form for *all* the sequences considered. Both valence and arousal ratings of all target stimuli—regardless of their valence category—were modified according to the valence of the preceding image. This was true only for emotional preceding images, however, replicating prior research; neurophysiological studies have shown that emotional but not indifferent preceding images influence electro-cortical responses to target images (Flaisch et al., 2008a,b), and report heightened emotional responses to stimuli with congruent emotional relative to indifferent contexts (i.e., N-N vs. I-N; P-P vs. I-P; Foti and Hajcak, 2008; MacNamara et al., 2009, 2011; but see Schupp et al., 2013).

Importantly, both indifferent and emotional targets were influenced by emotional preceding images. Further, no significant temporal-context effects were observed for indifferent targets in the random sequence, and the effects identified in the fixed sequences were comparable to that of emotional targets. This was contrary to our original expectations, and differs from the findings of previous studies (see Pastor et al., 2008; MacNamara et al., 2009, 2011). It is likely that this discrepancy emerged because, unlike previous studies, we examined all possible temporal context-target combinations. This included emotionally congruent (N-N, P-P) and incongruent context-target combinations (P-N, N-P), allowing us to explore the influence of negative context on positive targets and vice versa.

We also observed significant temporal-context effects for *both* emotional contrasts; that is, the influence of emotional temporal context was demonstrated in the ratings of target pictures preceded not only by negative but also positive images. This latter finding is contrary to the results obtained by Fujimura et al. (2013), who report that only negative preceding images influenced valence ratings of successive positive targets. We suggest that methodological differences may explain this inconsistency; while Fujimura et al. presented the same pictures repeatedly within varied contexts for a considerably longer period, we studied temporal-context effects across different images presented only once each. Therefore, our results indicate that positive temporal context also exerts a powerful influence on the evaluation of subsequent targets, even on those valenced negatively.

It is often reported that emotional responses to negative target stimuli are enhanced when preceded by images of the same valence (N-N; e.g., Wu et al., 2012). Although we did not observe such a congruency effect frequently in our data, when it did emerge within our fixed sequences it included negative targets predominantly. Recent neurophysiological research suggests that such discrepancies between positive and negative emotional stimuli reported frequently in the literature might be due to differences in the time-course of neurophysiological responses to these two categories, rather than differences between the stimuli themselves (see Weinberg et al., 2012). This potential neuroscientific explanation for the context effects we have revealed demands further investigation.

The second important finding to emerge from our study is that temporal-context effects manifest differently in different presentation sequences. When the same (fixed) randomized sequence was presented to the entire sample, temporal context exerted a large but transient effect that becomes progressively less pronounced over the course of the experiment. In contrast, within a random sequence generated separately for each participant, temporal-context effects were reduced yet consistent across trials. Notably, this modulating effect of sequence presentation emerged for emotional but not indifferent targets, and for *negative* target images especially. This finding was highly unexpected given the importance and biological relevance of negative stimuli in general, as indexed by the negativity bias phenomenon (e.g., Ito et al., 1998). As evidenced recently, however, the negativity bias is more likely to emerge under certain conditions (Weinberg et al., 2012; Hilgard et al., 2014); namely, within oddball rather than random or blocked presentation sequences, and for emotional stimuli that differ with respect to motivational relevance (Hilgard et al., 2014).

Furthermore, different sequences yielded different specific patterns of temporal-context effects. Although we observed assimilation (targets evaluated in line with the context) across the sequences more frequently than contrast (targets evaluated differently than their context), some temporal-context effects were reversed in sequences comprising only selected context-target combinations (e.g., only N-N and P-N). For example, the influence of positive context on negative target images (i.e., P-N) resulted in a contrast effect when only emotional context was available (P-N, N-N; i.e., *Fixed_INC_*). In contrast, when all three context categories were presented, the same combination resulted in assimilation (i.e., *Random_FIX_*). This diverges from the findings of Waugh et al. (2011). These authors presented a sequence of three same-valenced images in succession, however, which is likely to induce different degrees of emotional responses compared to our design. We interpret this result as an indication of heightened perceived similarity of context and target stimuli when fewer context categories are presented.

It is important to stress that these reversed context effects appeared primarily at the beginning of the fixed sequences, at which point target images within each valence category had similar normative ratings. On the basis of our own observations and the findings reported by other groups (Anokhin et al., 2006; Mormann et al., 2011; Sakaki et al., 2012; Kuhr et al., 2013) we suggest that maximal contrast effects at the beginning of fixed sequences might be a result of shared semantic content of such similarly valenced images. More systematic investigations into contrast effects are needed to determine the conditions that give rise to this specific contextual influence.

The third finding to emerge from our study also has considerable implications for emotion research; namely, the number of stimuli that precede a target image influences subjective ratings of the target. This Trial effect manifests as both habituation and sensitization, which were identified not only in fixed sequences whereby valence was manipulated artificially, but also in the random sequence. This latter observation shows that ratings change gradually over the course of an experiment, irrespective of the specific sequence employed. Moreover, the Trial effect modifies the influence of the immediately preceding image: Temporal Context-by-Trial interactions were revealed in all fixed sequences, resulting in only transient influences of preceding images. This stands in stark contrast to the random sequence, in which the influence of the preceding image persisted throughout the entire task. This might be the result of reduced variability in valence and arousal ratings for the fixed sequences, whereby all individuals viewed the same context-target combinations in the same succession.

The transient nature of these temporal-context effects and the large repetition of presented stimuli comprising typical neurophysiological studies might contribute to the inconsistencies concerning the effect of temporal context. Analyzing datasets without taking into account the time-course of context effects may result in non-significant overall context effects. This aspect should be considered while designing future studies.

Finally, we identified differences in temporal-context effects in terms of gender. In general, females appeared to be more sensitive than males to these confounding influences. This was the case especially when considering negative target images and preceding stimuli. Such results were expected on the basis of existing evidence (Gard and Kring, 2007; Lithari et al., 2010). Further, females were more vulnerable to design manipulations while evaluating the arousal of target images compared with males; specifically, when the Trial effect was introduced artificially in the *Fixed_INC_* and *Fixed_DEC_* sequences, females' arousal ratings decreased and increased, respectively, over the task more than males' for all target categories. This might be associated with known gender differences with respect to the relationship between valence and arousal in males and females in general (e.g., Bradley et al., 2001b); valence seems to be evaluated more independently from arousal in males relative to females. Interestingly, we also revealed opposing temporal-context effects, whereby females showed mostly contrast, while males expressed assimilation. This may indicate differences between genders regarding the conditions necessary for specific context effects to emerge.

The presence of gender effects reveals that the influence of temporal context manifests differently in males and females. This might indicate that temporal context manifests differently in other groups. Unfortunately we only collected demographic information from our sample, but future research could develop this finding by considering other groups defined more precisely, such as those defined according to personality, emotional and cognitive complexity, emotion regulation, or specific clinical samples. More emphasis should be placed on individual differences in future designs (Kuppens et al., 2013). Correspondingly, we agree with Hilgard et al. (2014) that statistical techniques which account for variability between individuals should be the first option when analyzing data from emotion research (e.g., GLMM).

## Conclusions

This study conducted the first comprehensive assessment of the influence of context effects on subjective evaluation of emotional images. Taken together, temporal-context effects were revealed in all presentation sequences, with valence and arousal ratings of negative target images being influenced most severely by temporal context. Although the random presentation sequence eliminated the temporal-context effect for indifferent target images, this was not true for negative targets and only partly true for positive targets. In contrast, temporal-context effects detected in the random-fixed sequence manifested in a transient manner, with habituation present for arousal ratings of emotional target images. Further, some of the temporal-context effects were reversed in sequences where not all possible context-target combinations were presented. Interestingly, females appeared to be more sensitive to these temporal-context effects than males. This is the case especially when we consider negative target and preceding images. Moreover, females seemed to be more vulnerable to the influence of design manipulations while evaluating the arousal of target images; ratings increased and decreased for the fixed sequences (*Fixed_INC_* and *Fixed_DEC_*, respectively) in all valence categories, for females but not males. Opposing directions of temporal-context effects within some of the sequences also indicate differences between genders—contrast effects were exhibited by females, while males expressed assimilation effects. We believe that these findings will contribute to future research on emotion and context. We have shown that it is more advantageous to employ fixed random rather than completely randomized sequences due to the transient nature of context effects they elicit. Introducing higher variability with completely randomized sequences does not eliminate context effects, while our artificial fixed sequences appeared to restrict these effects to the beginning of the task. It remains to be seen, however, whether the same findings are observed at other levels of emotion responding (e.g., neurophysiological).

### Conflict of interest statement

The authors declare that the research was conducted in the absence of any commercial or financial relationships that could be construed as a potential conflict of interest.

## Footnotes

^1^In order to simplify the presentation of the results, we refer to non-emotional (i.e. neutral) stimuli as “indifferent.”

^2^Numbers of employed IAPS photographs: Negative: 6200, 9452, 2753, 6561, 6213, 9290, 6571, 4664.2, 6550, 9432, 2750, 3550, 2710, 3220, 9600, 6312, 9520, 2730, 2900, 6260, 9500, 9420, 3181, 9911, 9300, 6370, 9421, 6540, 9560, 9220, 9910, 9253, 2205, 3030, 3350, 3301, 3110, 9040, 3168, 3015. Positive: 8130, 1610, 8162, 1510, 8034, 7480, 7350, 8180, 1340, 5001, 4640, 7280, 7260, 2331, 1500, 5270, 5470, 5200, 7230, 2345, 7580, 5780, 7270, 2311, 8470, 2170, 8380, 2216, 5600, 4290, 7200, 2209, 2352, 1610x, 8370, 2550, 1920, 5830, 5760, 2070. Indifferent: 5532, 8232, 2749, 7096, 4550, 7710, 7170, 2214, 7205, 4005, 2372, 4613, 4240, 7237, 5920, 7490, 7640, 7160, 2220, 4750, 9070, 4510, 7233, 7550, 8160, 7820, 5533, 4235, 2514, 2880, 2487, 7034, 7140, 7500, 4631, 7020, 2495, 4279, 7182, 7035.

^3^Stimulus order for the *Random_FIX_* sequence: I-P-I-P-N-P-I-P-P-N-N-I-P-N-I-N-N-N-P-N-P-N-N-N-P-P-P-I-N-N-N-I-N-N-P-I-P-I-N-N-P-N-I-N-I-N-P-I-P-N-N-P-I-I-I-P-I-N-P-N-N-P-I-N-N-N-I-I-N-P-P-P-N-I-N-I-N-P-I-P-N-P-P-N-P-I-I-P-N-P-P-P-N-I-I-P-I-I-I-P-I-P-I-I-P-P-I-I-N-I-I-P-N-I-I-N-P-I-P-I.
